# Vaccine Mandates in the COVID-19 Era: Changing Paradigm or Public Health Opportunity? Comment on "Convergence on Coercion: Functional and Political Pressures as Drivers of Global Childhood Vaccine Mandates"

**DOI:** 10.34172/ijhpm.2022.7616

**Published:** 2022-12-28

**Authors:** Anna Odone, Giulia Dallagiacoma, Giacomo Pietro Vigezzi

**Affiliations:** ^1^Department of Public Health Experimental and Forensic Medicine, University of Pavia, Pavia, Italy.; ^2^Collegio Ca’ della Paglia, Fondazione Ghislieri, Pavia, Italy.

**Keywords:** Vaccine Coverage, Mandatory Immunisation, COVID-19 Pandemic, Risk Perception, Public Health

## Abstract

The debate around vaccine mandates has flourished over the last decade, with several countries introducing or extending mandatory childhood vaccinations. In a recent study, Attwell and Hannah explore how functional and political pressures added to public health threats in selected countries, motivating governments to increase the coerciveness of their childhood vaccine regimes. In this commentary, we reflect on whether such model applies to the coronavirus disease 2019 (COVID-19) case and how the pandemic has re-shuffled the deck around vaccine mandates. We identify COVID-19 immunisation policies’ distinctive aspects as we make the case of countries implementing mass immunisation programmes while relying on digital COVID-19 certificates as an indirect form of mandate to increase vaccine uptake. We conclude by acknowledging that different forms of mandatory vaccination might serve as a shortcut to protect population health in times of emergency, underlining, however, that the ultimate public health goal is to promote voluntary, informed, and responsible adherence to preventive behaviours.

 The debate around vaccine mandates has flourished over the last decade, within and outside the scientific community, at the population and political levels, with several countries revising their vaccination policies in recent years, some introducing or extending mandatory vaccinations.^1^ Analysing the cases of Australia, California, Italy and France, Katie Attwell and Adam Hannah^2^ postulated that functional and political pressures added to public health threats and motivated governments to increase the coerciveness of their childhood vaccine regimes. Does this model apply to the coronavirus disease 2019 (COVID-19) case? How has the pandemic re-shuffled the deck around vaccine mandates?

 In general terms, mandatory immunisation represents the exercise of civil authority over individual judgment with the stated aim to protect public health. The concept of “mandatory vaccination policies” includes a wide range of aims and requirements of vaccination mandates, that are heterogeneous in terms of structure, exemptions, penalties, target populations and consequences for non-compliers.^3^ In 2021 we developed a conceptual framework of the spectrum of different immunisation mandatory approaches,^1^ which range from non-mandatory policies (ie, proactive voluntary policies) to mandatory policies with different degrees of enforcement (ie, mandatory policies without and with penalties, including denial of benefits, fines, job loss, custody loss and liberty penalties in a growing scale of severity), and varying exemptions schemes. The framework we proposed was developed on the one presented by Attwell and colleagues in 2018^4^ and converged on the responsibility that governments have to protect individual and population health. To schematise, we could say that the overall aim is to pursue collective health through population vaccine uptake and that all the different policies of the spectrum are means to reach such an aim. If that is the case, which setting-specific, epidemiological, functional or political elements determine what to opt for within the spectrum? Attwell and Hannah^2^ argue that more coercive approaches are enforced on political grounds and perceived by policy-makers to be “easier and cheaper,” as compared to other forms of intervention for increasing vaccination rates. Was this paradigm maintained during the COVID-19 pandemic? There are a number of distinctive elements to consider. The first element is the “objective epidemic risk” (as defined by Attwell and Hannah^2^) posed by COVID-19, an unprecedented health emergency associated with widespread infection transmission, morbidity and mortality; the second is population risk perception of COVID-19 disease, which heavily influenced – at different phases of the epidemic – population attitudes towards preventive behaviours and vaccines; and, third, large political pressures on governments responsible for controlling a pandemic causing massive health and socioeconomic impacts.

 Since they became available in December 2020, vaccines have been the most powerful tool to control the COVID-19 pandemic and mass vaccination campaigns were rapidly planned and implemented. As for COVID-19 containment and response measures in general, COVID-19 immunisation programmes and policies varied widely across territories and countries, and over time. Table reports COVID-19 vaccination mandates and provisions enforced in selected Western countries in the first phases of anti-SARS-CoV-2 (severe acute respiratory syndrome coronavirus 2) mass vaccination campaigns. Most of these mandates were eventually lifted after a period of time as high coverages were reached and pandemic control was improved. In most cases, vaccination was mandatory for selected groups of people, except in Austria, where it was extended to all subjects aged 18 years or more. Mandates targeted mostly healthcare workers,^5^ both at higher risk of infection and in closer contact with fragile and immunocompromised patients. Some countries, such as Canada and the United States, mainly targeted federal/state employees so as to ensure the health and safety of the state workforce and the efficiency of the civil service.

**Table:** COVID-19 Vaccination Mandates and Provisions Enforced in Selected Western Countries to Promote Vaccination Uptake (2021)

**Country**	**Target Group**	**Sanctions**	**Exemptions**
Australia	Healthcare workers in care homes for the elderly and in residential care	No data	Exemptions for healthcare workers varied by federal state/territory and can included medical exemptions
Austria	Citizens over 18 years of age	Fines from €600 to €3600	Medical (including pregnancy)
Canada*	Federal public servants in the Core Public Administration	Administrative leave without pay	
	Employees in federally regulated air, marine and rail transportation	Each organisation was required to guarantee employees were fully vaccinated. Staff fined $25 000 per violation	
	Travelers on federally regulated transport by air, rail and sea, aged 12+	No data	Emergency travel; medical exemptions
Germany	Healthcare workers and staff in care and medical establishments, including hospitals and care homes	No data	Medical
Italy	Citizens over 50 years of age	€100 fine; inability to access the workplace (no green pass)	Medical
	Healthcare workers and people who work in healthcare facilities	Employment suspension	Medical
The United Kingdom	Care home workers and anyone entering a care home	Redeployment	
The United States^a^	Federal workers and contractors of the federal government	Escalating up to dismissal	Religious and medical exemptions
	All service members of US armed forces	Administrative or non-judicial punishment	Weekly tests may replace vaccination
	Staff of Medicare- and Medicaid-certified healthcare providers	Possibility of sanctions	
	Staff of federally-controlled schools		Medical, religious, or administrative reasons
	Employers with 100 or more employees must ensure their workers are fully vaccinated		Weekly tests and personal protective equipment may replace vaccination

^a^Different provincial and local state governments put in place further requirements for mandatory vaccination (eg, in long-term care settings) and vaccine passports.

 Italy, first and heavily hit by the COVID-19 pandemic,^6^ and with its recent history of childhood immunisation mandates,^7,8^ was the first European country to adopt mandatory vaccination against COVID-19 for healthcare workers on April 1, 2021, with suspension without pay for non-compliers. Less than one year later, on January 8, 2022, a further vaccine mandate was imposed to all subjects over the age of 50, with a €100 fine and the denial of access to the workplace for those who refused to receive immunisation or failed to provide proof of recovery from COVID-19 (this measure was then relieved on June 15, 2022).

 While only a few countries in Europe imposed mandatory COVID-19 vaccination for the general adult population or selected high-risk groups, the digital COVID-19 certificate was required throughout Europe to travel, enter public places, restaurants, hospitals and offices, with requirements and limitations varying widely over time and across Member States. Again, Italy was among the European countries to impose the strictest rule on the digital COVID-19 certificate which was required to access the workplace. As we report, not only Italy imposed one of the strictest national-level stay-at-home orders in Europe, but also the strictest policies on direct and indirect COVID-19 immunisation mandates. Of particular interest is the “indirect” immunisation mandate obtained through extensive and long-lasting COVID-19 digital certificate requirements. Guided by both epidemiological and political pressures, such form of indirect vaccination mandate was basically extended to the whole eligible population and succeeded in pursuing high vaccination rates. In these respects, a crucial element was timing: the need to achieve extensive coverage in the shortest possible time to interrupt disease spread supported the adoption of stringent vaccination regimes.

 While we should update our conceptual framework by adding behavioural nudges,^9^ such as the COVID-19 digital certificate, as an intermediate strategy between non-mandatory voluntaristic and mandatory passive and punitive laws, we should reflect on its characteristics. If, on one side, asking COVID-19 digital certificate to let people in restaurants and bars was successful in making them get vaccinated (ie, the end justifies the means), on the other side, it did not make them understand the importance of getting vaccinated to protect their and others’ health. With the adoption of the European Union digital COVID-19 certificate, Europe resolved urgent systemic challenges, mediating between functional and political pressures.

 Another distinctive aspect of the COVID-19 pandemic has been the constantly evolving epidemiological scenario and associated media communication, which boostered the already volatile landscape of public sentiments towards vaccines, justifying the need to enforce coercive approaches,^10^ Albeit great risk perception of the disease possibly increased confidence in vaccines,^11^ major concerns arose at the population level about new vaccines’ safety and their effectiveness against, for instance, new SARS-CoV-2 variants. Indeed, the changing nature of the virus and the waning effectiveness of vaccines on immunity greatly affected public health goals of vaccination campaigns and policies over time^5^: especially with the emergence of Omicron, the general focus shifted from reaching herd immunity to reducing COVID-19 impact on overwhelmed healthcare systems, avoiding the further need for restrictive measures and protecting the most vulnerable subjects.

 The experience gained on vaccine policies in these difficult times must not be wasted or discouraged by further coercive measures, but rather reinforced by careful and consistent communication on the value and the limits of existing and future vaccines.^12^ Indeed, after the rollout of COVID-19 vaccines, several studies, conducted to explore the acceptance of mandatory vaccination policies, reported that mandates would be more likely to be accepted by individuals that willing to get vaccinated voluntarily against COVID-19. In particular, an experimental study conducted in Germany and the United States suggested that restricting people’s choices regarding vaccinations (ie, through compulsory policies) encourages respondents to act against such restrictions.^13^ Therefore, government requirements might strengthen anti-vaccine sentiment and political polarisation propagating vaccine scepticism, possibly undermining the acceptance of other vaccines.^3^

 Overall, despite the epidemiological and ethical justification of enforced mandatory vaccination programmes when the threat to public health is severe, their relatively easy nature and their proven effectiveness in counteracting unsatisfactory immunisation coverages and achieving optimal vaccination levels, it should be noted that coercive measures are rarely the solution to the root problem.^8^ They should be considered powerful but temporary tools implemented by governments to fulfil their responsibility to protect population health. On the contrary, the risk is that the original problem (ie, vaccination hesitancy) might be exacerbated through a loss of confidence in healthcare workers and policy-makers. More complex, but more effective strategies to tackle vaccination hesitancy should include comprehensive and persuasive communication campaigns aiming at educating the population without coercion. We acknowledge that direct and indirect mandatory immunisation is an effective paternalistic approach which should be considered in times of emergency to limit “objective epidemic risks,” together with alternative preventive measures, such as further lockdowns and other restrictions, just as divisive and undermining of trust in public health. Even so, we raise awareness on: (*i*) the need for governments to be credible and authoritative, with decision-making primarily based on scientific evidence adequately communicated to the general public.^14^ This helps to build trust and broader limits of acceptable government controls in case of emergency; (*ii*) the need for conducting continuous monitoring and evaluation of compliance to mandates and quantitative assessment on their impacts so as to optimise immunisation policies implementation; (*iii*) the need for direct public health and political commitment towards countering misinformation, educating target populations and training healthcare workers in order to maintain acceptable coverage rates, even without mandates.^15^

 The abundant available scholarly literature on mandatory vaccination, which has accumulated before and during the COVID-19 pandemic,^3,8^ clearly underlines the relevance of the topic but has not succeeded in disentangling its complexity related to ethical, juridical, public health and public policy considerations. As representatives of the public health community, we believe the COVID-19 mass vaccination experience should be capitalised on; it can teach us a lot on how to improve vaccination policies acting both on the demand and the supply side, and lessons learned can be adapted or scaled down to immunisation programmes other than COVID-19. With reference to mandatory approaches to vaccines, we learned that indirect forms of mandates might be effective in promoting population vaccine uptake and, in turn, protecting collective health. We knew from before that different forms of mandate increase immunisation coverage rates, but there is no evidence that they impact the demand-side determinants of vaccine uptake, nor that they influence confidence towards vaccines on the long run.^1^ Mandatory vaccination might serve as a shortcut to protect population health in times of emergency. Still, exploring determinants of vaccine confidence and investing in health education to promote voluntary vaccine uptake is much needed to enhance proactive uptake and support public trust. Promoting the individual and social value of immunisation through health education, advocacy, self-empowerment, and people-centred prevention high-quality services remains, therefore, the ultimate public health goal.

## Ethical issues

 Not applicable.

## Competing interests

 Authors declare that they have no competing interests.

## Authors’ contributions

 AO, together with GPV and GD, conceived the conceptual ideas. AO, together with GPV and GD, carried out the research. AO, together with GPV and GD, wrote the first draft of the manuscript and supervised the work. All authors provided important contributions to the interpretation of findings and contributed to the final version of the manuscript, and carefully revised and approved the final version of the manuscript.
